# Laughter and smiling facial expression modelling for the generation of virtual affective behavior

**DOI:** 10.1371/journal.pone.0251057

**Published:** 2021-05-12

**Authors:** Miquel Mascaró, Francisco J. Serón, Francisco J. Perales, Javier Varona, Ramon Mas

**Affiliations:** 1 Department of Mathematics and Computer Science, University of the Balearic Islands, Palma de Mallorca, Spain; 2 Department of Computer Science, Zaragoza University, Zaragoza, Spain; University of Tübingen, GERMANY

## Abstract

Laughter and smiling are significant facial expressions used in human to human communication. We present a computational model for the generation of facial expressions associated with laughter and smiling in order to facilitate the synthesis of such facial expressions in virtual characters. In addition, a new method to reproduce these types of laughter is proposed and validated using databases of generic and specific facial smile expressions. In particular, a proprietary database of laugh and smile expressions is also presented. This database lists the different types of classified and generated laughs presented in this work. The generated expressions are validated through a user study with 71 subjects, which concluded that the virtual character expressions built using the presented model are perceptually acceptable in quality and facial expression fidelity. Finally, for generalization purposes, an additional analysis shows that the results are independent of the type of virtual character’s appearance.

## Introduction

Facial expression modelling of virtual characters presents many difficulties, including time constraints, cost and complexity. On the one hand, from the field of psychology, approaches range from the pioneering work of Duchenne [1] and Darwin [2] to more recent contributions such as those of Provine [3], Gruner [4], Morreall [5] and Ruch [6]. These works address facial expressions and their relationship to human emotions. Ekman and Friesen published what they called Facial Action Coding System (FACS) [7], which has been used as a standard to categorize the facial expression of emotions. FACS has been employed by both psychologists and animators. On the other hand, from the field of artistic representation, most authors, such as Bridgman [8], Loomis [9], Hogarth [10] and Faigin [11], refer to the importance of knowledge of anatomy for proper representation of facial expressions.

In particular, laugh and smile are complex facial expressions. Both offers a great number of both emotional meanings and visual information. For example, the smile can have elements of other expressions such as sadness and anger, creating interesting effects of ambiguity and complexity. Therefore, the representation of laughter and smiling can be approached from different perspectives. In a previous work on laughter synthesis, DiLorenzo et al. [12] propose a physically based parametric model of the human chest that can be automatically driven from pre-recorded audio laugh samples. This model is anatomically inspired and synthesizes the movements of the torso muscle activated by the air flow within the body. The model is restricted to the respiration during the laughter act and it does not involve any facial motion. Cosker and Edge [13] propose another data-driven model for non-speech related articulations such as laughs, cries, yawns and sneezes. The model is based on a Hidden Markov Model (HMM) trained from motion-capture data and audio segments. Motion capture obtains the data using 30 markers placed on the face and normalized to a facial model. During training, this model learns the correlations between the recorded audio and the visual data. Griffin et al. [14] investigate how the laughter is perceived from body movements. But, they can only identify 5 different types of laughter.

From the virtual character modelling point of view, Niewiadomski and Pelachaud [15] consider how laughter intensity modulates facial motion. They focus on modelling both laughter and respiration. In their work, facial motions depends only on the laughter intensity but, different types of laughter are not considered. The same authors [16] also study the factors that influence the perception of AUs in virtual characters: Stimulus intensity, presentation (static or animated) and presence of wrinkles. In a first study, they evaluate the AUs of laughter, in terms of identification, naturalness and realism. A second study evaluates the expressions of laughter in terms of measuring the quality of the animation and its meaning. Also Niewiadomski et al. [17] do an experiment on how the intensity of the incongruity between audio and animation is perceived in an avatar with a laughing animation. The test assesses, among other things, its naturalness, plausibility and credibility. Urbain et al. [18] propose to compare the similarity of a sample with recorded laughter audio information and then select the corresponding sequence of facial expressions. The selection is done by measuring the acoustic similarities between the input laughter and the output one. Ding et al. [19] model hilarious laughter. They have developed a generator for face and body motions that takes as input the sequence of pseudo-phonemes of laughter and the time duration of each pseudo-phoneme. The generator learns the relationship between input signals and human motions. Then, it can be used to automatically produce laughter animation in real time. The same author [20] presents an animation controller of the upper body of a virtual character from audio input. Ochs et al. [21] identify the morphology and dynamic characteristics of different smiles in a virtual agent. They claim that there are three different types of smiles: funny, polite, and embarrassed. They create an algorithm and a web application to generate smiles with a virtual character. Mancini et al. [22] provide a virtual character with models of synthesis of laughter based on a paradigm of “expressivity-copying” and check how the presence of the character affects the perception of music and the mood of the user. Most of these works have a common approach: the integration of laughter into virtual characters (avatars) as a fundamental task for machine-human communication. This enables the design of sociable conversational agents using natural-looking and natural-sounding laughter. Unlike these previous works, the presented approach is only centred in the visual information (i.e., it does not depend on audio data).

Previous works focus on the generation of standalone valid models of laughter and smiles whilst our proposal is to develop a model within a general animation framework. To our knowledge, no references are found on laughter synthesis that focus on improving or creating specific tools for the character animation.

The structure of the paper is as follows. First, we present a computational model for the generation of facial expressions associated with laughter and smiling in order to facilitate the synthesis of such facial expressions in virtual characters. This model is based on the learning of real expressions by means of the facial feature tracking in video sequences. Next, by using the learnt animation parameters, we present the procedural animation system to reproduce different types of smiles, which are validated by conducting a user study. Finally, we finish by presenting the conclusions and discussing future work.

## Laugher and smiling modeling

In this section, we present a computational model for the generation of facial expressions associated with laughter and smiling. First, we define a taxonomy of laugh and smile. Next, we present the character’s facial model and how it is connected with a facial tracker in order to obtain the data for learning the human facial features’ motions. Finally, based on the obtained data set we describe a procedure to synthesize such facial expressions in virtual characters.

### A taxonomy

**Fig 1:**
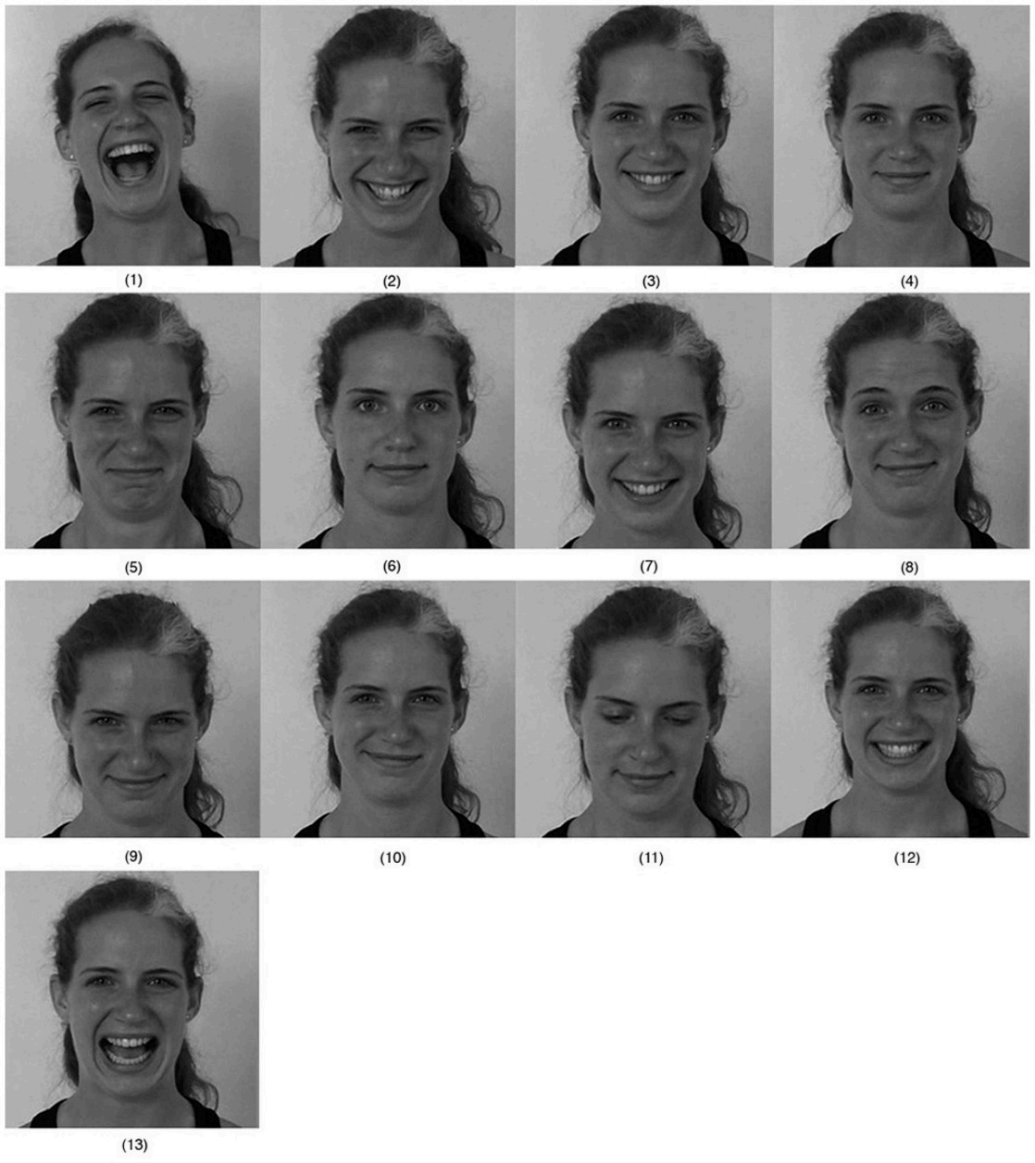
Laugh and smile taxonomy based on the different expressions of joy.

First, we present a taxonomy to classify the different types of laugh and smile. Earlier laugh and smile classifications come from the field of psychology, and their main interest is to ascertain the expression’s authenticity. In this category are the works of Ekman [6, 23–25]. From these works, smiling and laughter are claimed to be universal indicators of joy [26]. From the expression synthesis point of view, we refer to the work of Faigin [11], where it can be found an exhaustive taxonomy of different types of expressions of joy (see Fig 1).

This taxonomy is based on the observation of separate key elements in laughter and smiles and how they work. First, it present a classification according to the joy intensities, going from *laughter* to *smile* (Fig 1, from 1 to 4). To this classification, it is possible to add the results of combining the actions of the zygomatic and orbicularis muscles with different positions of the eyebrows, corresponding to the forms defined in the universal expressions. Thus, the *sly smile* combines a smile with eyebrows of the anger expression (Fig 1.9); *avid laugh* with surprise expression (Fig 1.7); the *ingratiating smile* with the expression of fear (Fig 1.8) and the *melancholy smile* with the sadness expression (Fig 1.6). Other categories involve voluntary expressions: the *stifled smile* (Fig 1.5) is produced to suppress the spontaneous laughter, and the *abashed smile* (Fig 1.11) is produced to repress satisfaction. The *debauched smile* (Fig 1.10) is the combination of the smile with a position of the eyelids in which the pupils are partly covered; it is the position that can be associated with states of drowsiness and intoxication. Finally, the taxonomy is completed by the *false smile* and *false laughter* (Fig 1.12 and 1.13).

### Facial model

**Fig 2:**
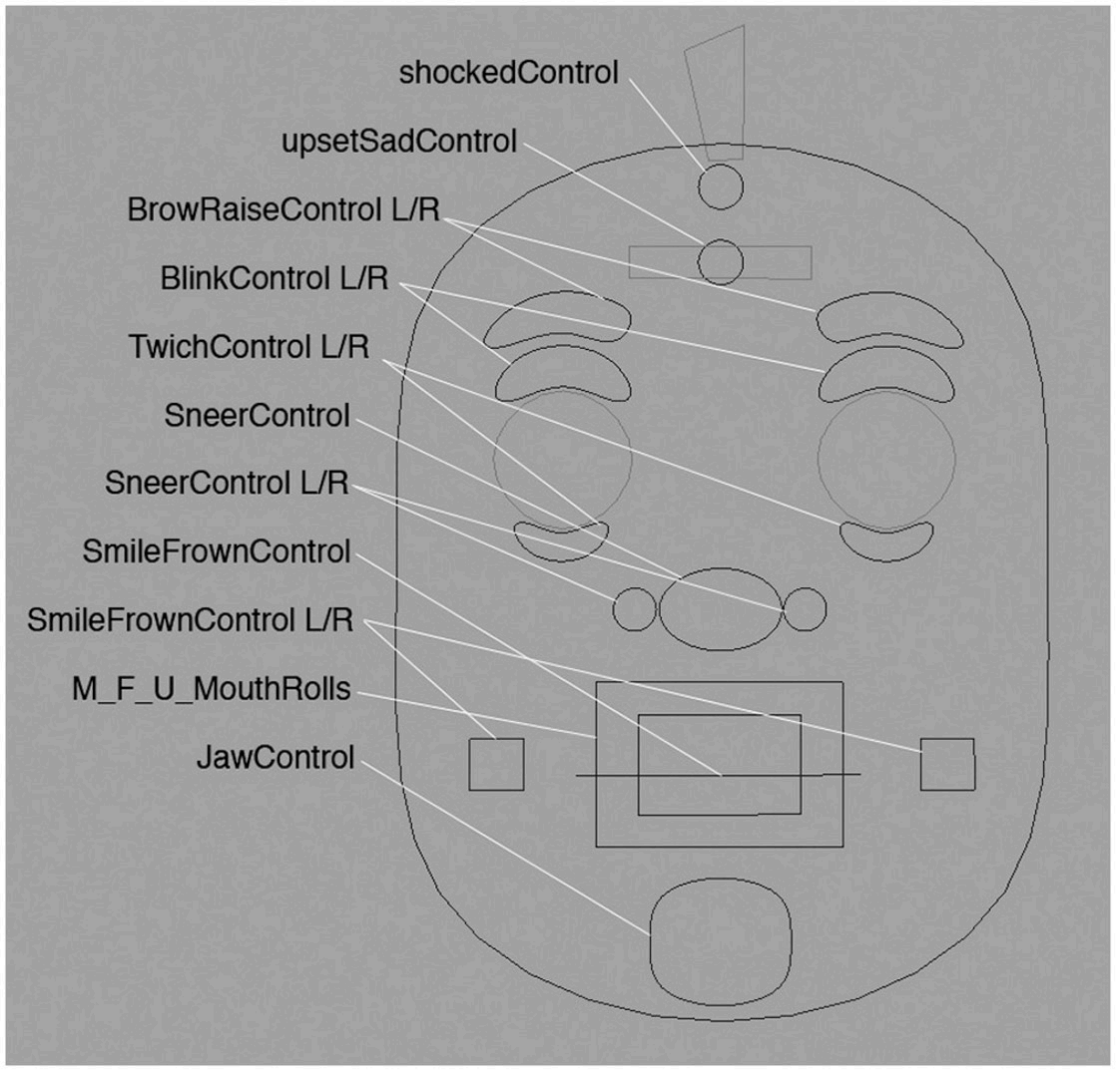
Rig controllers.

A virtual character’s facial model must allow the representation of diverse faces in both realistic and cartoon aesthetics while retaining its anthropomorphic properties. Facial rigging is the process of defining the animation controls for a facial model. In this regard, the virtual character’s rig should provide a method to faithfully reproduce all facial muscle activity. For this reason, the character’s rig has been designed using the edge-loop technique [27]. This technique optimizes the facial deformations distributing the geometry as muscle lines. There is no standard for defining the rig system interface; in our case, we have chosen an interface based on a 3D view with 2D handlers, similar to the solutions proposed by Alexander et al. [28] and Digital tutors [29]. With these approximations, we have an exhaustive control of the geometric deformation that performs the mesh simulation of facial muscles as well as an intuitive tool to easily perform all the facial actions. In conclusion, we use a general-purpose rig that, in addition to helping in the design of any facial expression, can be used specifically to describe laughter and smiles (see Fig 2). To define the deformations of the character meshes, our rig uses a combination of techniques for bone and blendshape interpolation. Thus, the user is able to control hundreds of polygons to generate all the facial expressions with the simplified use of animation curves. These animation curves automatically drive all the rig controllers.

**Fig 3:**
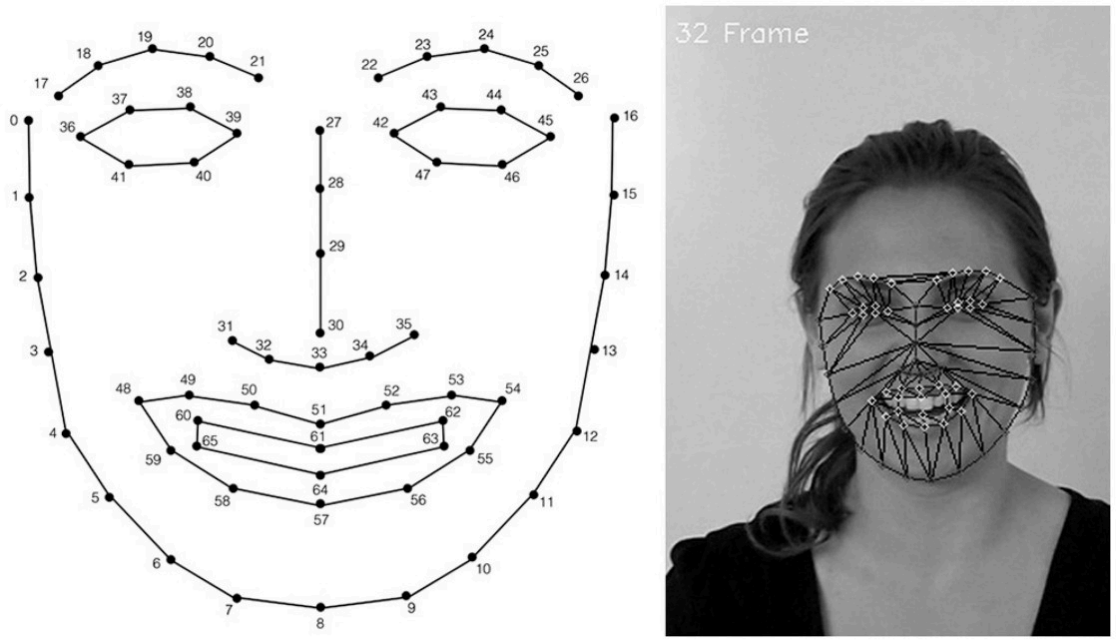
Visual tracking of facial landmarks.

In order to model the animation curves for each laugh and smile facial expression, we want to learn the real facial features’ motions associated with these expressions. To avoid the use of special hardware, learning is based on the computer vision algorithm of Saragih et al. [30]. By means of this algorithm, a 66-point mesh of facial landmarks is obtained for each sequence frame, which is tracked following the motion of the user’s motions (see Fig 3).

A temporal rule-based method [31] is used to set the facial rig animation curves from the tracked facial landmarks. First, in Table 1 we define the model facial parameters *FP* = {*FP*_1_, *FP*_2_, …,*FP*_15_}. Next, correspondences from facial parameters to facial landmarks (*P*_*i*_, where 0 ≤ *i* ≤ 65) and controller actions (*C*_*i*_, where 1 ≤ *i* ≤ 17) are defined in Table 2.

**Table 1:** Facial parameter definition.

FPs	Action
*FP*_1_	Half opening of lower lid (right)
*FP*_2_	Internal distance between eyebrow and eyelid (right)
*FP*_3_	Half opening of lower lid (left)
*FP*_4_	Internal distance between eyebrow and eyelid (right)
*FP*_5_	Distance of eye opening (right)
*FP*_6_	Distance of eye opening (left)
*FP*_7_	Width of mouth
*FP*_8_	Distance corner of mouth to nose (right)
*FP*_9_	Distance corner of mouth to nose (left)
*FP*_10_	Height of mouth
*FP*_11_	Vertical between mouth and nose
*FP*_12_	Distance between corner of mouth and nose-wing (right)
*FP*_13_	Distance between corner of mouth and nose-wing (left)
*FP*_14_	Vertical between chin and nose
*FP*_15_	Distance between oval of face

**Table 2:** Facial landmarks and rig controllers’ correspondences.

FPs	Landmarks	Rig controller
*FP*_1_	46–47	*rightTwich*
*FP*_2_	22–27	*upsetSad, shocked, rightBrownRaise*
*FP*_3_	40–41	*leftTwich*
*FP*_4_	21–27	*upsetSad, shocked, leftBrownRaise*
*FP*_5_	22–42	*rightBlink*
*FP*_6_	21–39	*leftBlink*
*FP*_7_	48–54	*smileFrown*
*FP*_8_	33–54	*rightSmileFrown*
*FP*_9_	33–48	*leftSmileFrown*
*FP*_10_	51–57	*jaw*
*FP*_11_	51–53	*sneer*
*FP*_12_	35–54	*rightSneer*
*FP*_13_	31–48	*leftSneer*
*FP*_14_	8–33	*jaw*
*FP*_15_	0–16	*neck*

**Fig 4:**
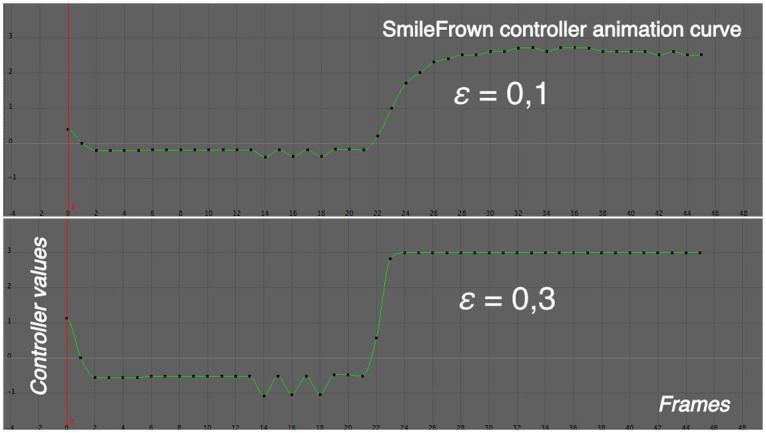
Animation curve for the *SmileFrown* controller at different *ε* values.

Therefore, we have defined a direct correlation between the facial points’ motions and the values that the rig controllers must take to generate the desired facial expression. Thus, for a frame *i*, the controller action value, *C*_*i*_, is given by Eq (1)

$$\begin{array}{c} C_{i}=\left(FP_{x_{i}}-FP_{x_{0}}\right)\cdot \varepsilon , \end{array}$$ (1)

where *FP*_*x*_ is the specific facial parameter defined in Table 1, the subscript 0 refers to the neutral expression, and *ε* is the controller adjustment factor. This factor depends on the animator, who is responsible for choosing the hardness or softness of an expression (affecting on the degree of realism he wants to obtain). Empirically, we found that visually correct values of *ε* may range from 0.1 to 0.3 for a 640x490 face image resolution corresponding to a subject’s frontal plane. For instance, Fig 4 shows the animation curve for the *SmileFrown* controller of the *Smiling Open-Mouthed* expression. The x-axis represents time (frame) and the y-axis represents the controller’s values. The scaled curve at a value of *ε* = 0.1 is shown at the top. At the bottom is represented the curve adjusted for *ε* = 0.3. It is possible to observe that this parameter controls the expression smoothing between realistic values.

By means of these transformations, our system generates a curve for each controller action from a video sequence containing the desired facial expression of laugh and smile.

### Laugh and smile learning

In order to learn the animation curves to create realistic expressions that involve laugh and smile, we employ different facial expression data sets with the types of laughter and smiles defined in the previous taxonomy.

Classical databases of facial expressions such as **CK+** [32] and **MMI** [33] include expressions that are all conveniently annotated and include the main emotions. In particular, there are data sets for detection and classification of types of laughter as the **BBC Smile Dataset** [34], the **Uva-Nemo** database [35], which was generated to study the dynamics of a spontaneous smile and a voluntarily generated smile, and the **MAHNOB Laughter database** [36], which has been used for the differentiation and detection of laughter and smiling during speech sessions, Haddad et al. [37] present the AmuS database which is a database for the synthesis and recognition of joy in speech (English and French). It is primarily a study of speech acoustics, distinguishing between smiled-speech and speech-laughs. Jansen [38] has created MULAI which is a database that classifies types of social laughter based on their social function. It includes recordings of dyadic interactions (two subjects) and contains body movement data, ECG (electrocardiogram) and GSR (galvanic skin response). However, these databases do not address other types of laughter than the associated with expressions of joy. Therefore, regarding the used taxonomy, in previous databases there are representative examples of *uproarious laughter*, *laughter*, *open-mouth smile*, *closed-mouth smile*, *false smile* and *false laugh*, but not of others. For this reason, we have built a new facial expression data set, the **MASEPESmile** database, which provides exactly the 13 types of laughter described in the taxonomy.

The **MASEPESmile** data set (freely accessible from http://ugivia.uib.es/MASEPESmile/) consists of 91 videos of seven subjects, with acting experience. The subjects has given written informed consent (as outlined in PLOS consent form) and they know that their participation is voluntary, and that their images will be used for research purposes, assuring that their privacy, anonymity, and confidentiality will be protected.

**Fig 5:**
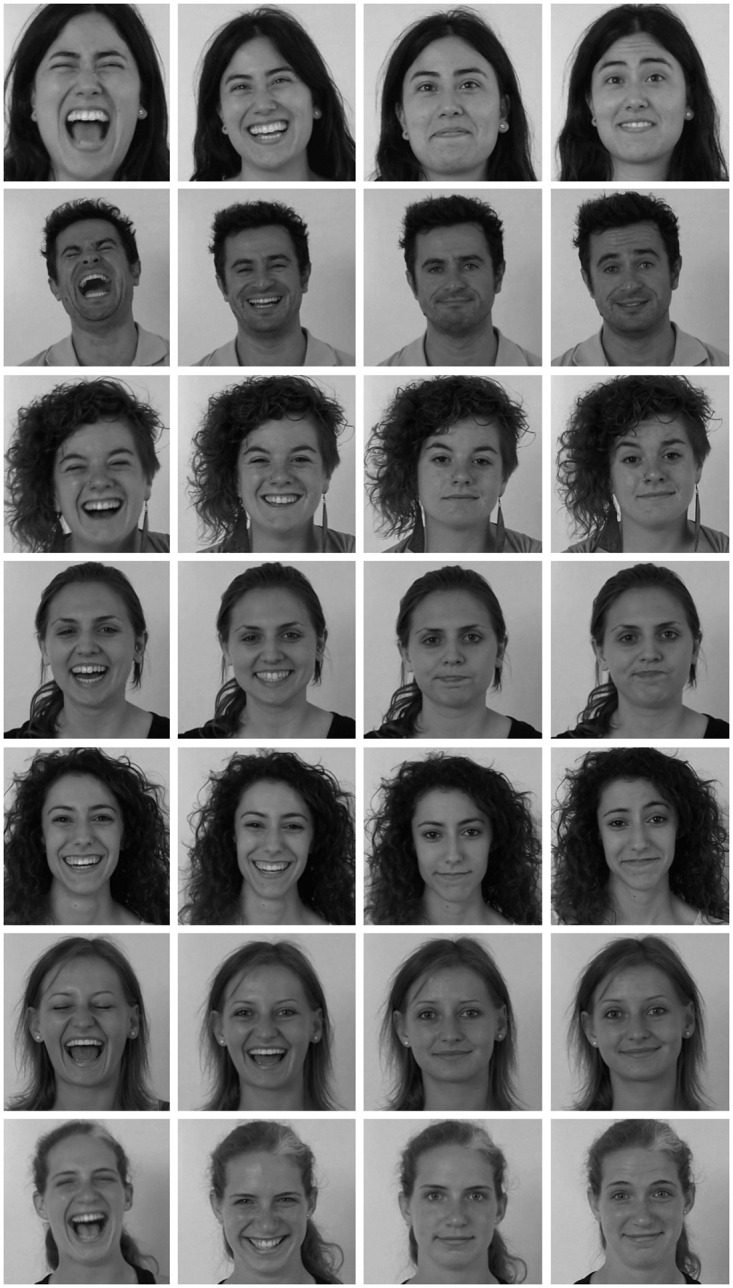
Multiple subjects with different laughter and smiling types of the MASEPESmile data set.

The data set recording was done in a room with controlled lighting. Each actor entered the recording set individually in order to minimize the influence of the other actors participating in the recording. Before each recording, the director described in detail the expression to perform. For example, for the expression *Sly Smile*: “It is the laughter of the evil one, of the astute one, of the paid one of itself, the eyebrows rise from the outside and wrinkle in the middle, the eyelids swell with pressure at the bottom and the lips are thin and stretch upwards pressing along the skull.” In addition, in front of the actor was projected an image with a picture of the expression taken from Faigin’s work [11]. Once ensured the actor understood what he has to perform, the expression was recorded. Finally, the expression was validated by a human expert and the recording is repeated as many times as necessary to meet the smile requirements. Each subject was asked to perform the 13 types of laugh and smile, starting at the neutral expression. Fig 5 shows 7 subjects of the data set performing four of the 13 categorized expressions. Specifically and ordered by columns we can see: *uproarious laughter*, *smiling open mouthed*, *melancholy smile* and *ingratiating smile*.

**Fig 6:**
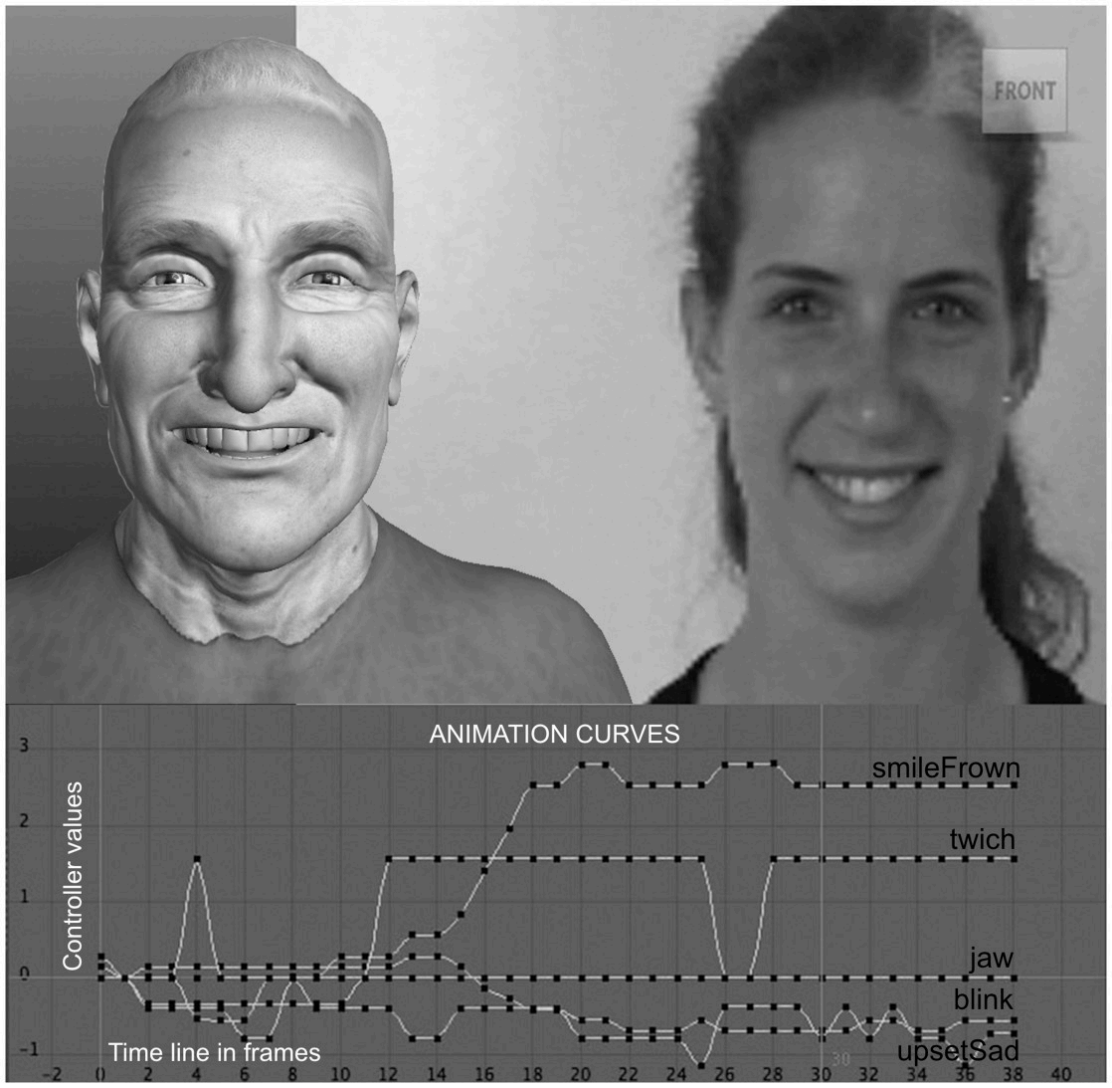
Mapping data for expression *open-mouth smile* to a virtual character.

In previous section is detailed how to process one frame to compute one controller curve’s point given the landmarks (see Eq 1 and Tables 1 and 2). Then, it is possible to compute the animation curve for each sequence of the MAPESmile data set. For instance, in Fig 6 is shown a virtual character animation key frame, the controllers’ animation curves, and the original video frame of expression *open-mouth smile*.

**Fig 7:**
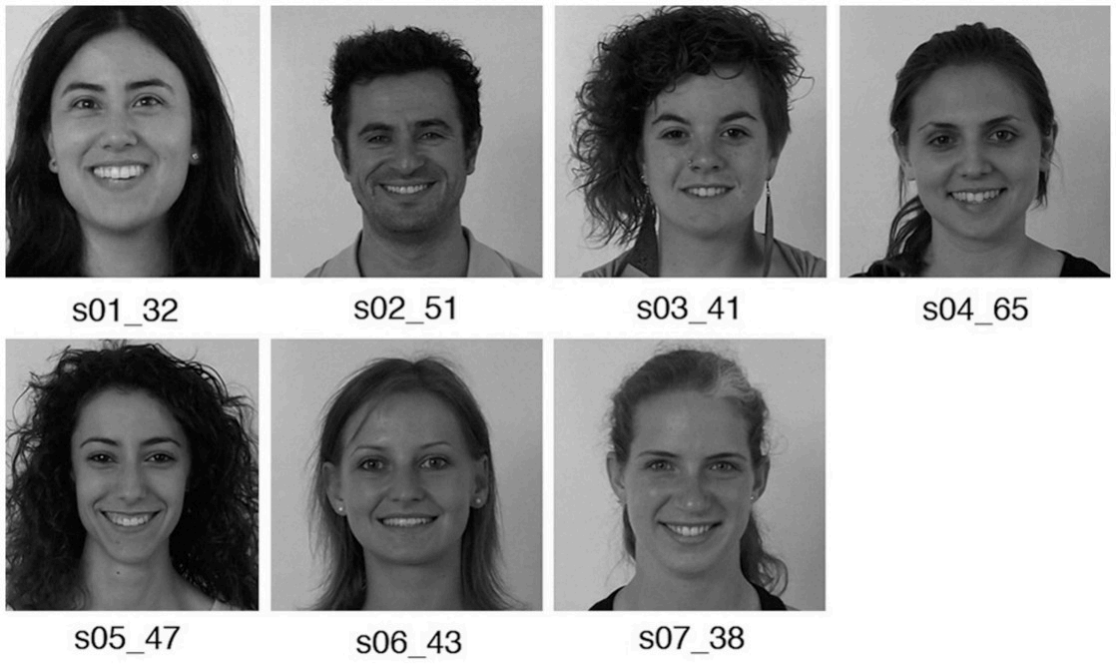
Open-mouth smile performed by different subjects.

Once computed the animation curves for all the data set sequences, following the previously explained procedure for each sequence, it is possible to establish the most representative curves for the animation of all the considered types of laugh and smile. These most representative curves will allow the generation of a procedural animation system for reproducing different types of laugh and smile of the data set. In order to learn the most representative animation curves, first, we apply dynamic time warping to find the optimal alignment between each controller values. Thus, we can determine the similarities between different facial expression performances. For instance, for the expression *open-mouth smile*, recorded by the seven subjects of our experiment (Fig 7), in Table 3 are shown the alignment values for the *smileFrow* controller.

**Table 3:** Distance alignment values for the *smileFrown* controller for the *open-mouth smile* expression.

	*s01*	*s02*	*s03*	*s04*	*s05*	*s06*	*s07*
*s01*		12.85	12.88	27.82	16.68	36.82	11.31
*s02*	12.85		42.15	10.90	8.66	16.17	9.16
*s03*	12.88	42.15		48.25	35.20	38.21	18.76
*s04*	27.82	10.90	48.25		4.78	4.29	13.42
*s05*	16.68	8.66	35.20	4.78		9.56	12.43
*s06*	36.82	16.17	38.21	4.29	9.56		13.66
*s07*	11.31	9.16	18.16	13.42	12.43	13.66	
*mean*	19.73	16.65	32.57	18.24	14.55	19.79	13.12
*standard deviation*	10.32	12.79	13.82	17.00	10.87	14.32	3.21

This process is extended to all controllers and all the laugh and smile facial expressions in order to learn the representative curves. For each subject, we apply the K-means algorithm using the alignment value as distance between each data set sample. The resulting performance prototypes are selected as the most representative animation curves. Finally, the desired facial expression animation is obtained by applying the most representative curves to the facial model. Thus, newly generated expressions take into account the different performances included in the data set to generalize the virtual character animation curves for the synthesis of new laugh and smile facial expressions. Examples of the results are shown in the videos included in the supporting information.

In addition, by analyzing the alignment tables for different expressions and controllers, it is possible to explain the obtained expression models. For instance, in the case of the expression of *uproarious laughter*, the minimum alignment distance for the *smileFrown* controller has a huge value (42.38), which indicates that several data set expressions are less generalizable due to a subject’s performance dependency. But, in these cases, the cycle of laughter is more prevalent. This fact, is demonstrated in the work of Ruch [6], which describes how the cycle basically depends on the lung volume, which obviously depends on the subject.

## Results and discussion

### User study: Expression description

For the data set recording, prior to perform an expression, the actor receive a description of a context where such an expression would occur and its corresponding classification. A user study was conducted in order to validate these descriptions used to build the MAPESmile data set. In addition, with this user study, we can compare the perception of the real data set expressions with the generated virtual expressions.

Fifty-one unpaid students of both sexes aged between twenty and twenty-eight years old were recruited. All the participants has given written informed consent (as outlined in PLOS consent form) prior to testing to ensure that they know that their participation is voluntary, that they will incur no physical or psychological harm, that they can withdraw at any time, and that their privacy, anonymity, and confidentiality will be protected.

In order to conduct the experiment, each participant rank six or seven expressions randomly chosen from the taxonomy (approximately the 50% of the expressions). For each chosen expression, first, the participant sees the description of the expression employed to built the data set. Next, the participant sees the two videos of the real actor and the virtual character of the selected expression, and answers the question: *Does the expression corresponds to the description?*. This question is scored on a scale ranging from 0 (very little) to 5 (very much).

**Fig 8:**
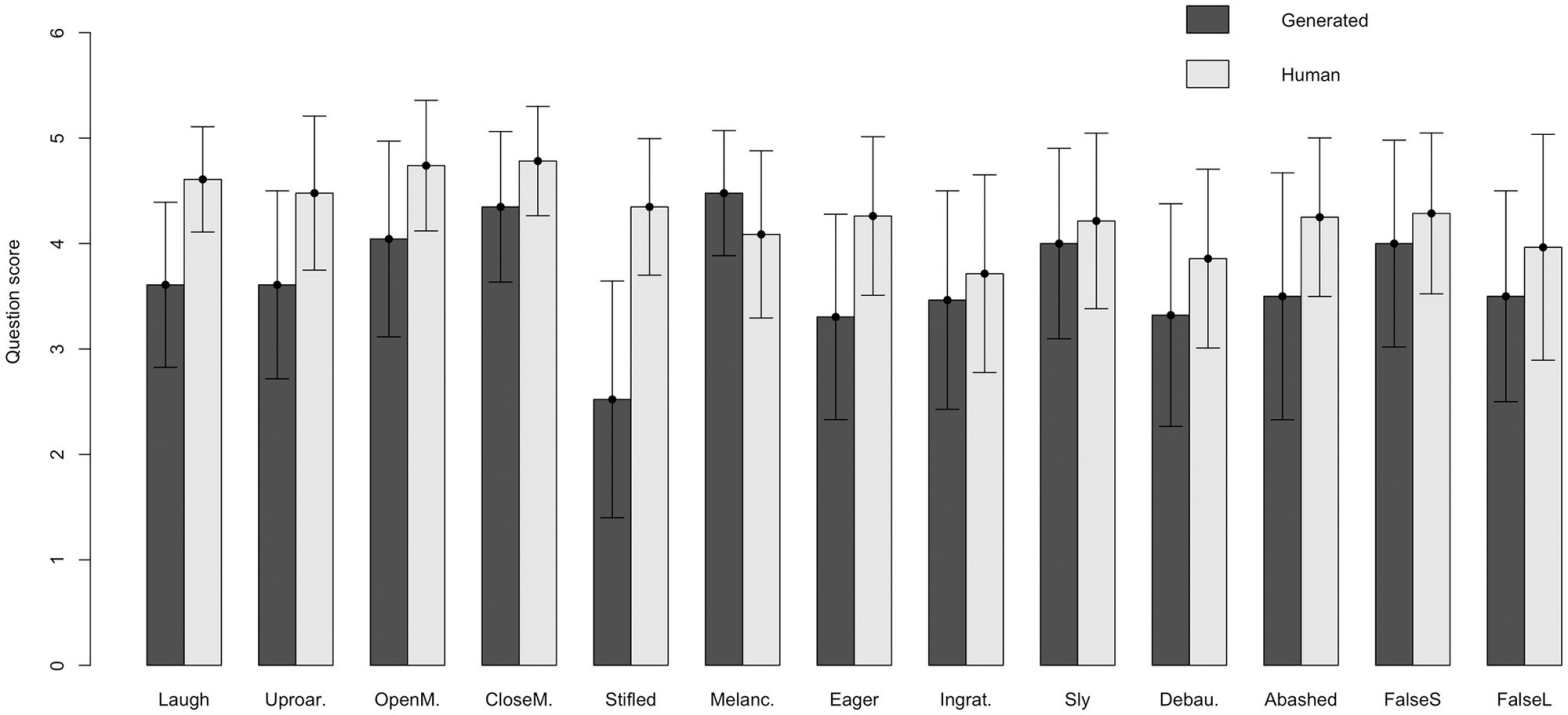
Comparison of the question scores for each expression performance (real vs generated).

As expected, it can be observed in Fig 8 that real expression are better perceived than the generated virtual expression. The grand mean for the real expression was 4.27 (SD = 0.32). For the generated virtual expression, the grand mean was 3.4 (SD = 1.09). Then, the answers of the question show that the generated virtual character expressions corresponds to their descriptions.

### User study: Movement quality and representativeness

**Fig 9:**
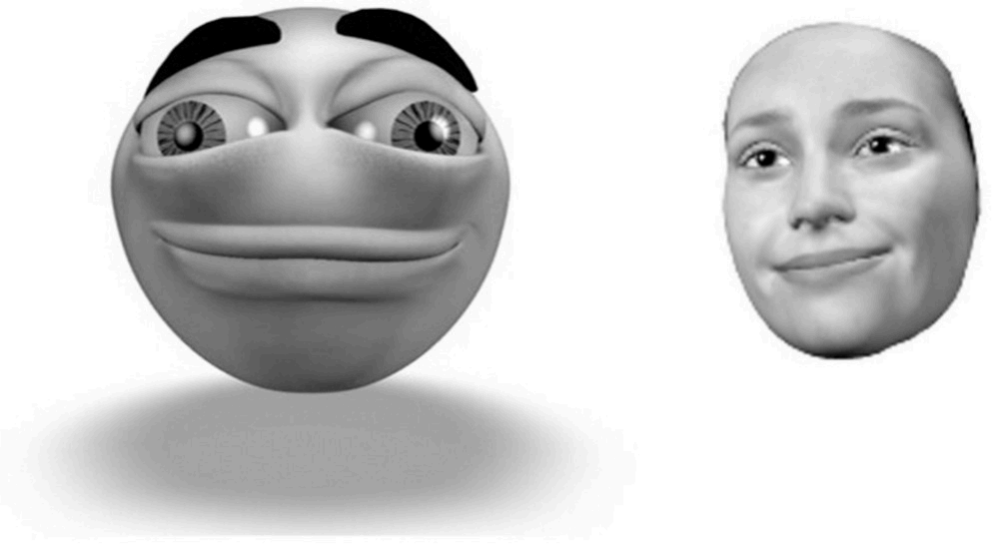
Character avatar synthesis for the *melancholy smile*.

To validate our laugh and smile model, we design a user study for a human evaluation of the movement quality and representativeness of the generated expressions in one avatar. In addition, we are interested in studying whether the avatar used could influence the user perceptions. We used two different avatars: a realistic avatar, and a cartoon avatar (see Fig 9). The expressions of both avatars are made with the same animation curves, synthesized by applying our previously described method.

In order to study the perception of the synthesized virtual character expressions, seventy-one unpaid university students of both sexes aged between nineteen and twenty-one years old were recruited (a within-subject design was used). All the participants has given written informed consent (as outlined in PLOS consent form) prior to testing to ensure that they know that their participation is voluntary, that they will incur no physical or psychological harm, that they can withdraw at any time, and that their privacy, anonymity, and confidentiality will be protected. Therefore, each participant seeing thirteen videos of virtual character facial expressions, one for each expression in our study. After seeing each video, they answered two questions:

- Q1: Does the motion feel right?
- Q2: Does the motion represent the description?

The objective of Q1 is to test the expression realism; meanwhile, the goal of Q2 is to validate the perceived expression.

Both questions are answered on a sheet where a brief description of the expression will be found. Each question is scored on a scale ranging from 0 (very little) to 5 (very much).

**Fig 10:**
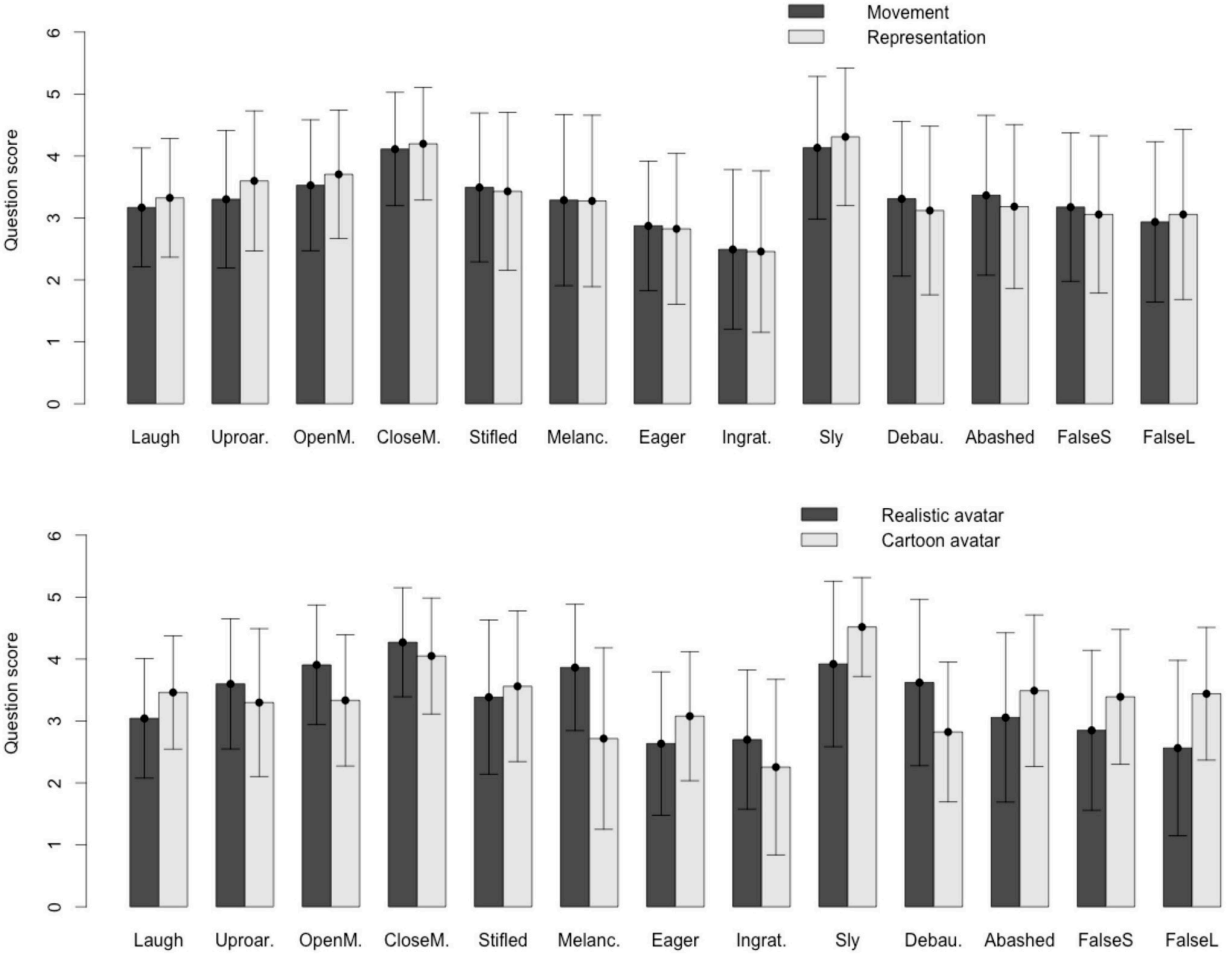
Mean and standard deviation (+/- SD) of the scores for both questions for all the evaluated expressions (71x13x2 = 1846 scores and 71x13x4 = 3692 scores).

The grand mean for Q1 was 3.32 (SD = 0.45). For Q2, the grand mean was 3.34 (SD = 0.51). Thus, the test answers show that the generated expressions were realistic and that they were positively perceived by the users. Nevertheless, it can be observed in Fig 10 that the users did not perceive all the expressions in the same manner. The best-scoring expression is sly smile (M = 4.13 and SD = 1.14 for movement; and M = 4.30 and SD = 1.11 for representativeness), followed by the smile with mouth closed (M = 4.11 and SD = 0.91 for movement; and M = 4.19 and SD = 0.90 for representativeness). The worst-scoring expression is ingratiating smile (M = 2.49 and SD = 1.28 for movement; and M = 2.45 and SD = 1.30 for representativeness), followed by eager smile (M = 2.87 and SD = 1.04 for movement; and M = 2.82 and SD = 1.21 for representativeness).

There is a distinction between the highest-scoring expressions and those rated worse. We think that among the latter are those whose meaning can be more confusing, while the meanings of the top-rated expressions are less ambiguous. Thus, *ingratiating smile* in the test has been described as *suggestive* or *to get sympathy*, and *eager smile* has been described as *anxious* or *euphoric*, which received the worst scores, coinciding with the ambiguity in their descriptions.

By analyzing the scores of both questions by the kind of avatar used to represent the expression, we found that the grand mean for the realistic avatar was 3.34 (SD = 0.56) and that for the cartoon avatar was 3.33 (SD = 0.56).

With two-way ANOVA, we found that the main effect of the avatar on the score was not statistically significant (*F*(1,3640) = 2.825, *p* > 0.05). The results by avatar and expression are shown in Fig 10.

There was a significant expression interaction effect (*F*(12,3640) = 5.167, *p* < 0.001), which was due to the difference between the two avatar expressions of *melancholy smile* and *false laughter*, as determined by a Scheffé post hoc analysis. In the *melancholy smile* case, the realistic avatar has better scores, while for the *false laughter* expression, the cartoon avatar is better perceived. This fact is explained if we consider that *melancholy smile* is the combination of facial action units corresponding to two opposite emotions such as joy and sadness, so, this expression is one of the most complex to interpret, especially when out of context, as in our test. It is reasonable that the representation of this expression by the realistic avatar has a better acceptance because the realism of the avatar facilitates the understanding and validation of the movement. In the case of *false laughter*, we should realize that this expression is the one with the largest interpretative load, not only because it is consciously performed but also because it has a voluntary component of exaggeration. This is what is expected from all the expressions of cartoon characters and what explains the result obtained.

Summarizing, from the analysis of the user study, we can conclude that the expressions generated using the presented method are acceptable in movement quality and in fidelity of representation of expressions, and this result is independent of the type of avatar used; that is, the expressions generated by the presented system are accepted independently of whether they are performed by a realistic or by an unrealistic character, as it follows the anthropomorphic rules.

### Limitations

Our model is versatile and adapts to most facial animation rigs, as long as the isolated animation of the movement of the eyebrows, eyelids and mouth accepts the animation keys that our system generates. Of course, is a requirement for the animation environment to accept the .anim format of the Autodesk Maya software. Another constraint of our model is that it is not currently integrated as a plugin within the animation environment, this can be a drawback as the user needs to manually import the curves into their scene.

## Conclusions and future work

In this work, we defined a framework capable of producing realistic representations of the different types of laughter and smile in virtual characters. The presented model is systematic, automatic, and it is generalizable to different characters.

A video data set has been generated with thirteen expressions involving laughter or smile, which follows the presented taxonomy.

For the generation of the automatic animation of the facial rig controllers, we have provided a facial motion capture-based system, which converts these data into information for the animation rig. The integration of this information into the animation environment allows it to be further refined and modified by the animator. We have presented a rigging system for control of geometric interpolation of the twenty-six polygonal surfaces, which correspond to the muscle actions and are capable of being combined to form AUs, as described by Ekman and Friesen in the work on FACS [7].

The method followed to generate facial animation has been extensively applied to our data set, to choose the most representative controller’s animation curve for each expression. This choice has been made using dynamic time warping and the K-means algorithm. Therefore, our study provides a library of representative animation curves to animate expressions of laughter and smile on virtual characters, improving the efficiency of the animation process.

To validate the resulting virtual character animations, we have conducted a test on rating of the quality of the motion and of the expression representativeness. The obtained results validate our proposal.

From the presented framework, different lines of investigation have been opened, which could define various extensions of the work. First, in order to improve the obtained results, the data set could be extended by recording and analysing the laughter and smile expressions of a greater number of individuals. In addition, the presented framework could be employed to model other facial expressions.

## Supporting information

S1 Video Uproarious laughther. (M4V)

S2 Video Laughter. (M4V)

S3 Video Similing open-mouthed. (M4V)

S4 Video Similing closed-mouth. (M4V)

S5 Video Stifled smile. (M4V)

S6 Video Melancholy smile. (M4V)

S7 Video Eager smile. (M4V)

S8 Video Ingratiating smile. (M4V)

S9 Video Sly smile. (M4V)

S10 Video Debauched smile. (M4V)

S11 Video Closed-eye (abashed) smile. (M4V)

S12 Video False smile. (M4V)

S13 Video False laughter. (M4V)
